# Reversible switching mode change in Ta_2_O_5_-based resistive switching memory (ReRAM)

**DOI:** 10.1038/s41598-020-68211-y

**Published:** 2020-07-09

**Authors:** Taeyoon Kim, Heerak Son, Inho Kim, Jaewook Kim, Suyoun Lee, Jong Keuk Park, Joon Young Kwak, Jongkil Park, YeonJoo Jeong

**Affiliations:** Electronic Materials Research Center, Korea Institute of Science and Technology, Seoul, South Korea

**Keywords:** Electronic devices, Electrical and electronic engineering

## Abstract

We report the complementary resistive switching (CRS) behaviors in a tantalum-oxide based resistive switching memory device that reversibly changes its switching mode between bipolar switching (BRS) and CRS in a single memory cell depending on the operation (compliance current) and fabrication (oxygen scavenger layer thickness) conditions. In addition, the origin of the switching mode transition was investigated through electrical and optical measurement, where the conductance is believed to be determined by two factors: formation of conductive filament and modulation of Schottky barrier. This result helps design a resistive switching device with desirable and stable switching behavior.

## Introduction

Resistive random access memory (ReRAM) having a simple metal–insulator–metal (MIM) device structure is considered one of the promising future device for various applications such as nonvolatile data storage^1^, in-memory logic^2^, and neuromorphic devices^3^. The devices utilize ion migration in combination with internal redox reaction^4^, leading to formation and rupture of conductive filament, and consequently change the resistance of switching layer. Many studies have reported attractive properties of ReRAM: high density integration, long-term retention time, small size, and fast switching speed^4,5^.

Two main types of ReRAM are conductive-bridge RAM (CBRAM) and valance-change memory (VCM) depending on what materials are involved in the resistance switching^6,7^. In VCM, oxygen ions or oxygen vacancies are widely accepted as a movable ion species forming a conductive filament and various transition metal oxides, e.g. Ta_2_O_5_^8^, HfO_2_^9^, and TiO_2_^10^, have successfully provided a role of host material for the ion migration and conductance change. Among them, Ta_2_O_5_-based ReRAM using bilayer structure with oxygen scavenger or reservoir layers, e.g. Ta^11^ or Ti^12^ or TaO_x_^13^, have reported excellent performance with stable bipolar resistive switching.

In the meantime, ReRAM can exhibit three different switching behaviors such as unipolar, bipolar, and complementary resistive switching (URS, BRS, CRS respectively)^14,15^. As named, URS is termed for a device using the same voltage polarity for Set and Reset respectively, whereas BRS devices require opposite bias polarities for the two operations. In contrast, CRS ReRAM can be simply made by staking two BRS ReRAM cells anti-serially^16^ and thus, one of the connected devices is subjected to Set operation while the other one is supposed be Reset under the same voltage applied. This allows great advantage to the CRS, reducing leakage current through the passive devices significantly, since one of the stacked ReRAM cell is always in the OFF state regardless of the actual device state^17^. As a result, it can solve the sneak path current problem, which results in disturbance in the output current levels during read operation and also causes insufficient voltage or current driving from the peripheral circuit during program mode in a large scale array without using selector device^18,19^. Despite of these benefits, complicated device structure than typical BRS ReRAM and destructive read operation make it hard to be implemented practically^14^. It has also been reported that in some cases CRS behavior appears unintentionally in a common single device without back-to-back stacking^20^ and this can hinder the expected usage of the bipolar memory device.

In this study, we found that a resistive switching mode in a single Ta_2_O_5_-based ReRAM can reversibly change between BRS and CRS operation depending on operation condition: high current at programming leads to clear CRS behavior and it is more severe as reducing the scavenger (or reservoir) layer thickness. We analyzed the switching mode change by comparing previously reported CRS mechanisms in conjunction with the measured electrical and optical data such as the Ultraviolet (UV) transmittance and Ultraviolet Photoelectron Spectroscopy (UPS). Oxygen ions in Ta_2_O_5_ switching layer after creating oxygen vacancy may penetrate into Ta scavenger layer and combine with Ta metal and create tantalum sub-oxide. With high programming current and thin Ta layer, entire oxygen concentration in Ta film increases and this ends up leading to the switching mode transition to CRS by modulating Schottky barrier height at the interface between Ta and Pt. This work helps understand switching behavior of Ta_2_O_5_-based bilayer ReRAM and maintains stable operations.

## Results and discussion

### Resistive switching behavior of Ta_2_O_5−x_ based ReRAM

**Figure 1:**
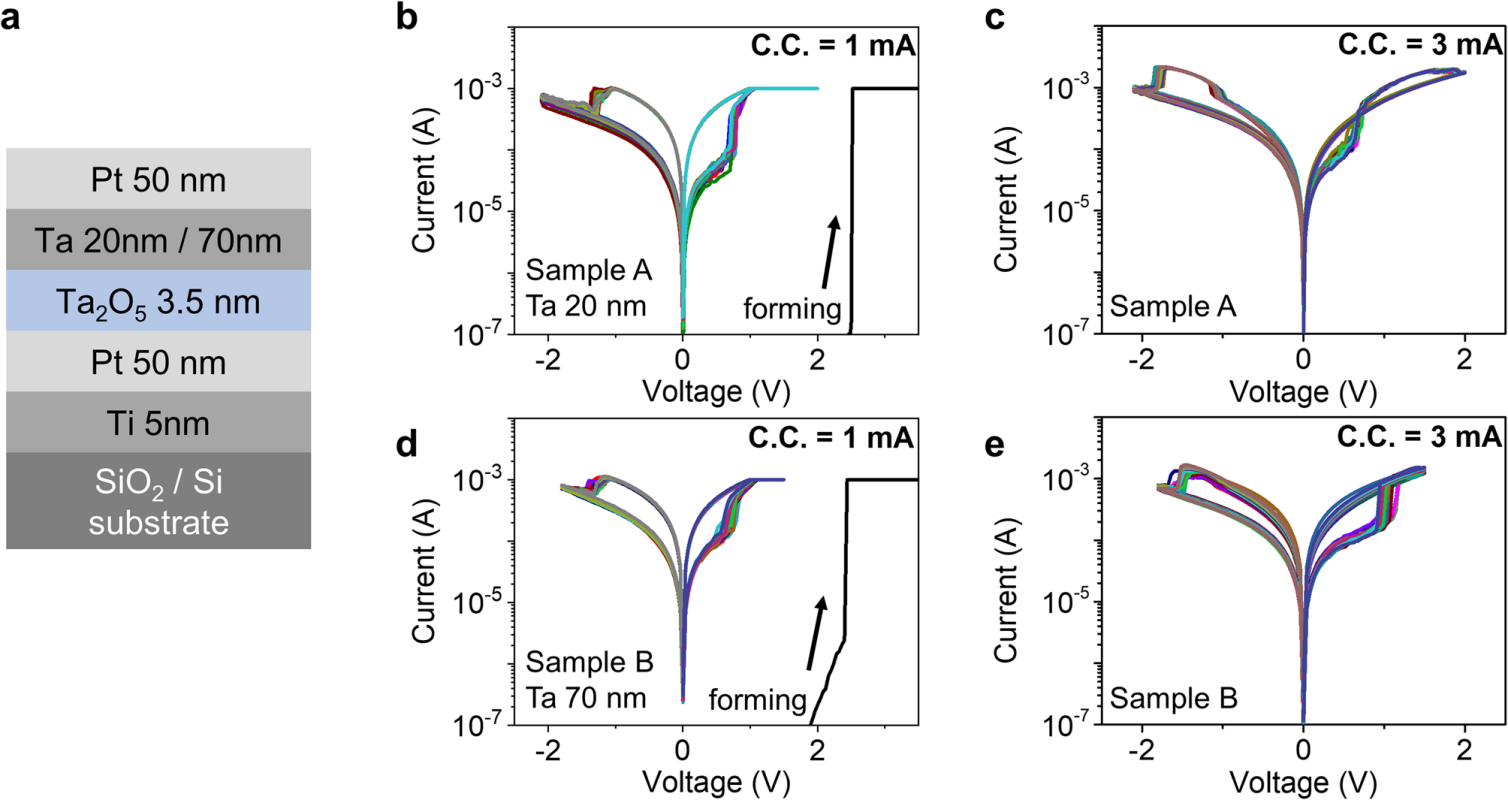
Schematic diagram of Ta_2_O_5_-based RRAM device and *I*–*V* curves (**a**) schematic of Ti/Pt/Ta_2_O_5_/Ta/Pt structure with Ta-Pt top electrode and Pt bottom electrode. Tantalum layer under Pt-TE is (**b**), (**c**) 20 nm and (**d**), (**e**) 70 nm, respectively. The forming voltage (V_form_) in both samples is about 2.5 V. The complimentary resistive switching type only appears when applying higher compliance current to a device having thin Ta layer.

**Figure 2:**
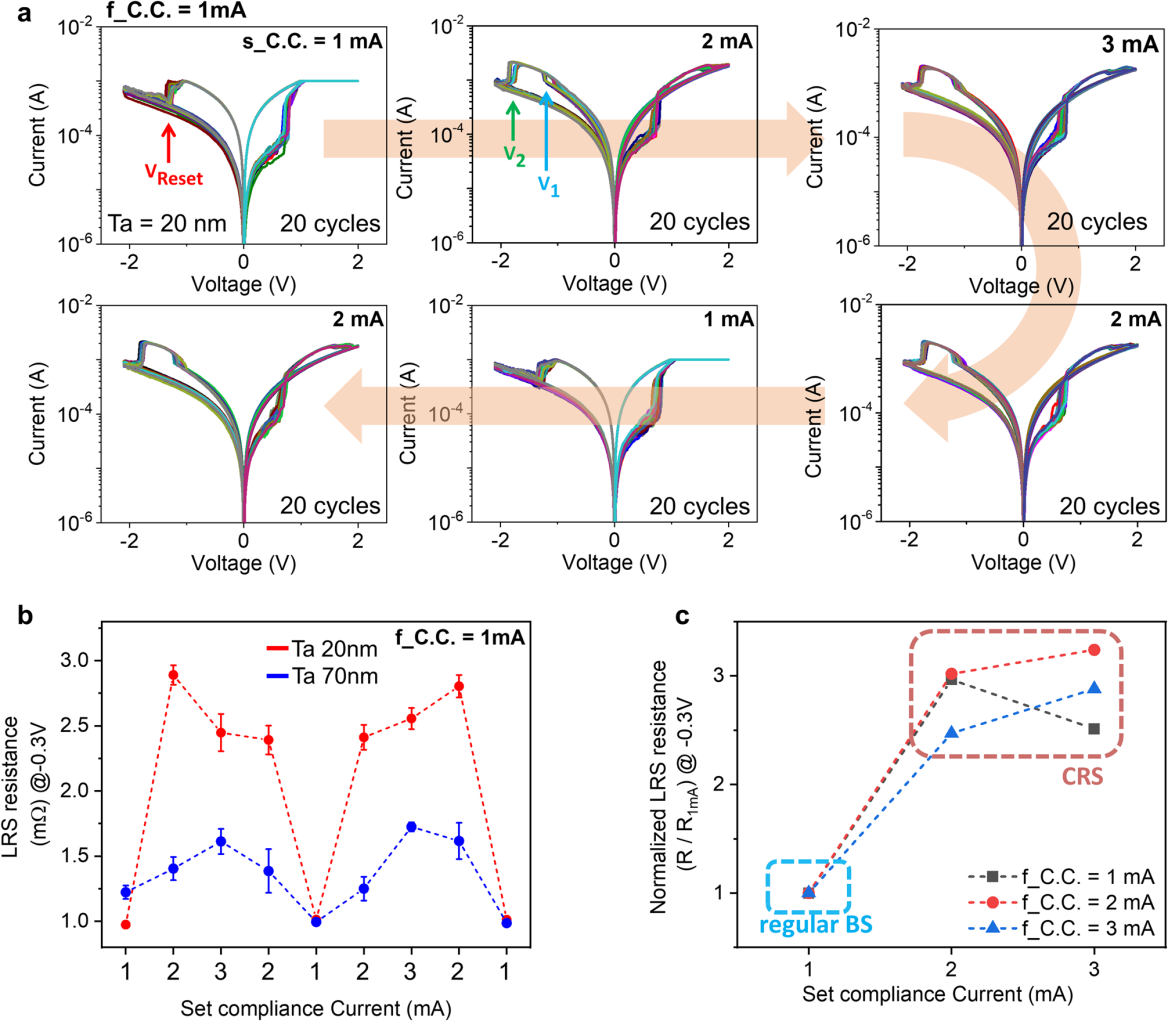
DC current–voltage characteristics of Pt top electrode devices (**a**) Ta 20 nm thickness device showing switching mode change from BRS at low C.C. to CRS when increasing C.C. levels. (**b**) LRS resistance plots of two samples (Ta 20 nm and 70 nm) by sequentially changing C.C. In Ta 20 nm device, LRS level clearly varies indicating switching mode change depending on C.C., while the thicker Ta device maintains LRS level. (**c**) LRS resistance plot with different forming compliance current (f_C.C.) from 1 to 3 mA.

Two samples with different Ta film thickness were prepared: sample A: Pt(50)/Ta(20)/Ta_2_O_5_(3.5)/Pt(50)/Ti(5) and sample B: Pt(50)/Ta(70)/Ta_2_O_5_(3.5)/Pt(50)/Ti(5), where the numbers are thickness of each layer in nanometers. The bottom electrode was formed by photo-lithography and lift-off process using radio-frequency (RF) sputtering of Ti and Pt on commercial SiO_2_/Si substrate flowing 20 sccm of Ar gas. Following Ta_2_O_5_ oxide, resistive switching layer, was deposited by RF sputtering with a Ta_2_O_5_ ceramic target with low power (30 W)^21^. After that, Ta and Pt electrodes were also formed by RF magnetron sputtering and photo-lithography process. The device has cross-point structure with cell size 8 $\upmu \mathrm{m}$ × 8 $\upmu \mathrm{m}$ and the stack is shown in Fig. 1a. The *I*–*V* curve of the fabricated devices were measured using a Keithley 4200 semiconductor parameter analyzer (Keithley 4200 SPA, Keithley Instruments, Inc.). Figure 1b–e show different resistive switching behaviors of fabricated devices depending on Ta layer thickness and current compliance (C.C.) levels at Set operation. For reliable results, the measurements were repeated 20 times for each condition. Under low C.C. (1 mA in Fig. 1b, d), standard bipolar switching (BRS) was manifest in both sample A and B, while the switching behavior was separated in the two samples at higher C.C. (3 mA in Fig. 1c, e): sample A showing CRS switching and sample B maintaining the BRS mode. In a nutshell, the switching mode of the fabricated Ta_2_O_5_-based ReRAM varied depending on the measurement condition and device structure, where CRS type appears only when applying higher C.C. to a device having thin Ta layer. Interestingly, two switching modes was observed in one device, sample A, and they changed to the other one reversibly as shown in Fig. 2a. Here, C.C. levels of Set operation were changed sequentially by repeating increase and decrease: 1 mA, 2 mA, 3 mA, 2 mA, 1 mA, and 2 mA. Again, the 20 cycles of *I*–*V* curves were measured at each C.C. level and it moved to the next C.C.. When C.C. changes from 1 to 2 mA, the switching mode changed from BRS to CRS and after that, BRS was recalled again if C.C. was reduced from 2 to 1 mA. Therefore, one can tune the switching mode whenever required by simply modifying C.C. In Fig. 2b, the sequential mode change are summarized by monitoring the resistance of low resistance state (LRS) at − 0.3 V of read voltage since the LRS level shows more distinctive change than high resistance state (HRS) for the switching mode changes. In sample A (red dotted line), the CRS mode represented by higher LRS level at 2 mA and 3 mA C.C. evidently differs from low LRS level of BRS at 1 mA C.C. In contrast, sample B with thicker Ta film (blue dotted line) has much less dependency of LRS level and keeps the bipolar operation despite of the varying C.C. levels. Figure 2c shows similar LRS trends with Fig. 2b regardless of the forming compliance current (f_C.C.). The resistances of 2 mA and 3 mA s_C.C. in Fig. 2b varies mainly due to the stochastic filament forming and rupturing process, but the resistance values showing CRS behavior (2 mA and 3 mA) are always higher than the BRS mode (1 mA).

### Models for CRS behavior in Ta_2_O_5_-based ReRAM

**Figure 3:**
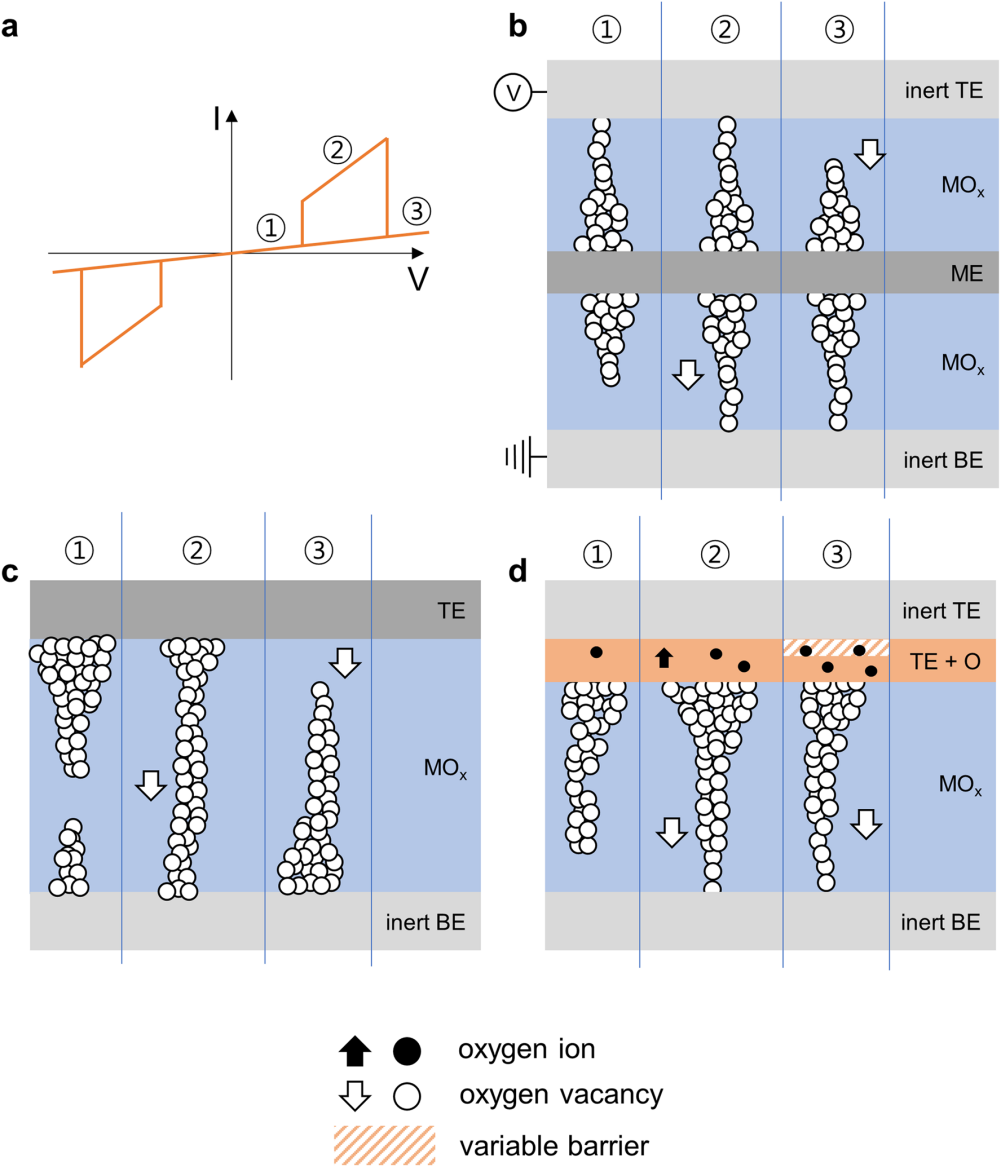
Possible Complementary Resistive Switch (CRS) mechanisms (**a**) *I*–*V* curve of common CRS device. Schematic illustration of complementary resistive switching processes (**b**) in two anti-serially connected switching elements. (**c**) In a single bipolar cell with insufficient number of movable ions. (**d**) In a single bipolar cell with active TE metal electrode. The cell has variable barrier (oxide/TE or inert TE). The white arrow and circle indicate location and movement of oxygen vacancies. And black arrow and circle indicate location and movement of oxygen ions.

The typical *I*–*V* curve of CRS shown in Fig. 3a can be divided into three regions and several models have been proposed to describe the mechanism. In this paper, we assumed that voltage bias is applied to the top electrode. One of the well-known case is that two bipolar type devices are connected anti-serially as shown in Fig. 3b^22^. At first, in region 1, the upper cell between top electrode (TE) and middle electrode (ME) is in the LRS state and the lower cell between ME and bottom electrode (BE) has the HRS state, thus the current is suppressed. By applying higher positive bias, oxygen vacancies migrate to the BE direction and this leads to the formation of conductive filament in the lower cell, while the upper one is still maintaining the LRS state. Thus, in the region 2 (Fig. 3a), both devices are in the LRS state; as a result, much higher current can flow through the device. However, with further higher positive bias, conductive filament in the upper cell starts to rupture. Hence, in region 3, the device shows the similar low current level with region 1 despite of the different position of filament gap inside the switching layer. The model can depict CRS behavior well, but being not applicable to our Ta_2_O_5_-based device having just a single device stack in Fig. 1a. The second model is the case that the number of movable ions in the switching layer is insufficient to build a stable conductive filament^20^. The limited number of ions generally results in making weak filament as a consequence, and thus, after completing a filament in Fig. 3c (region 2), the additional ion migration by higher applied voltage causes rupturing of the already formed conductive path from the filament base (TE side in region 3). Though the model works in a single device stack case like our Ta_2_O_5_ ReRAM, it does not fully account for the experimental switching properties. In a common ReRAM, higher C.C. level allows stronger filament requiring high V_Reset_ to erase the Set state. However, under the postulated condition, limited movable ions, formation of stronger filament and larger V_Reset_ for erase is not necessarily true when applying larger C.C. Rather, it could result in weaker filament due to ion distribution. Based on this, the clearly higher CRS turn-off voltage, V_2_ in Fig. 2a, than BRS V_Reset_ in 1 mA C.C., is not accountable by using the second model when ions are insufficient. More evidently, since the model is based on the total amount of ions, when the number of movable ions varies, the device should give different switching behavior. However, in Fig. 2c, Ta_2_O_5_-based device keeps showing the similar CRS behavior even changing C.C. level of device forming process (f_C.C.), which is believed to affect the amount of the movable ions. This indicates the second model does not cover our device as well.

The next model takes into consideration of Schottky barrier height modulation at the TE interface as a source of CRS behavior^23^. In Fig. 3d, under positive bias, two competing effects influencing device conductance take place together: one is formation of filament in the oxide layer reducing device resistance and the other is oxidation of the scavenger layer (Ta) due to oxygen ion penetration. The later can make the device highly resistive (HRS) by raising Schottky barrier at the TE interface neither conductive filament exist in the switching layer (Ta_2_O_5_) or not. Thus, the device changes to HRS state even after building a filament and shows CRS behavior if the Ta layer is oxidized as shown in Fig. 3d.

### Measurement of band structure and discussion

**Figure 4:**
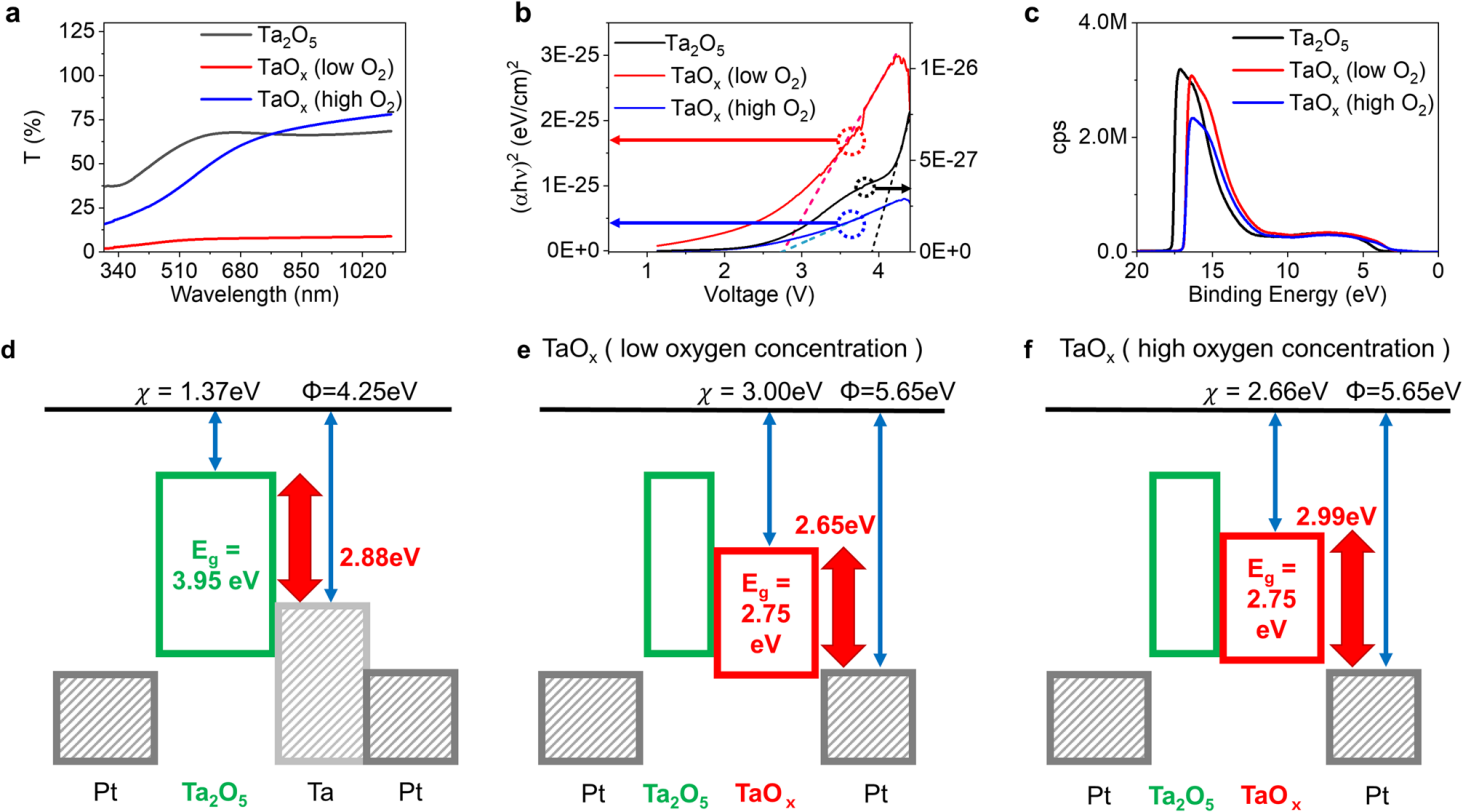
Measurement of band structure (**a**) Transmittance plot and (**b**) Tuac relation plot using transmittance and (**c**) UPS result of Ta_2_O_5_, TaO_x_ (low O_2_) and TaO_x_ (high O_2_) layer. The band structure of the Pt/Ta_2_O_5_/Ta/Pt device as shown (**d**), (**e**), (**f**). Each figure represented in (**d**) as-fabricated device, and device with (**e**) TaO_x_ (low oxygen concentration) and (**f**) TaO_x_ (high oxygen concentration).

**Figure 5:**
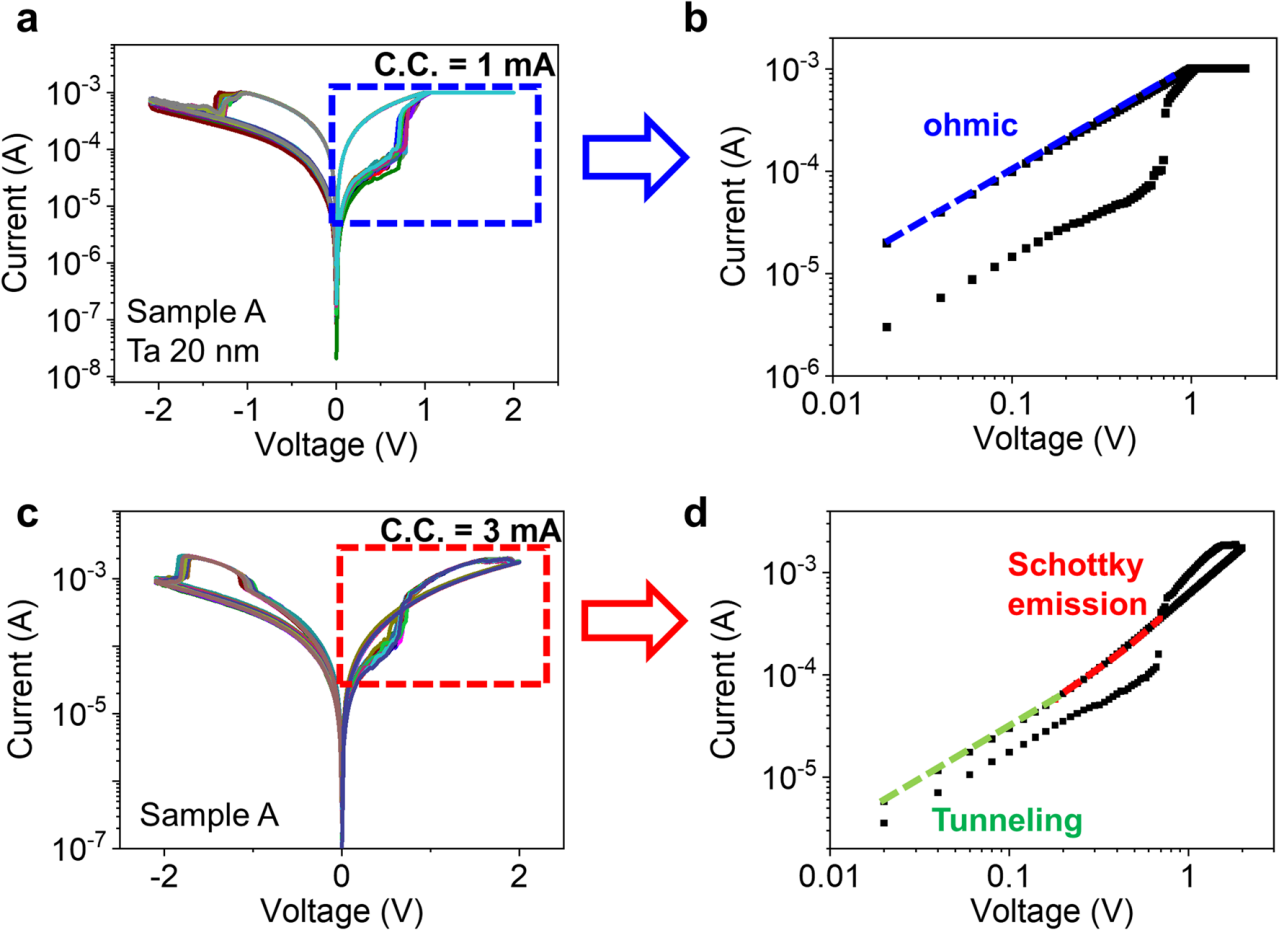
Resistive switching conduction mechanism (**a**) *I–V* characteristics of Pt/Ta(20 nm)/Ta_2_O_5_/Pt device with set C.C. = 1 mA (**b**) Fitting positive voltage *I–V* data (Sample A, C.C. = 1 mA) for the nonlinear *I–V* curve (**c**) *I–V* characteristics of Pt/Ta(20 nm)/Ta_2_O_5_/Pt device with set C.C. = 3 mA (**d**) Fitting positive voltage *I–V* data (Sample A, C.C. = 3 mA) for the nonlinear *I–V* curve: Tunneling conduction at low voltage; Schottky emission at HRS (high voltage). The fitting results are highlighted by color dash line.

**Figure 6:**
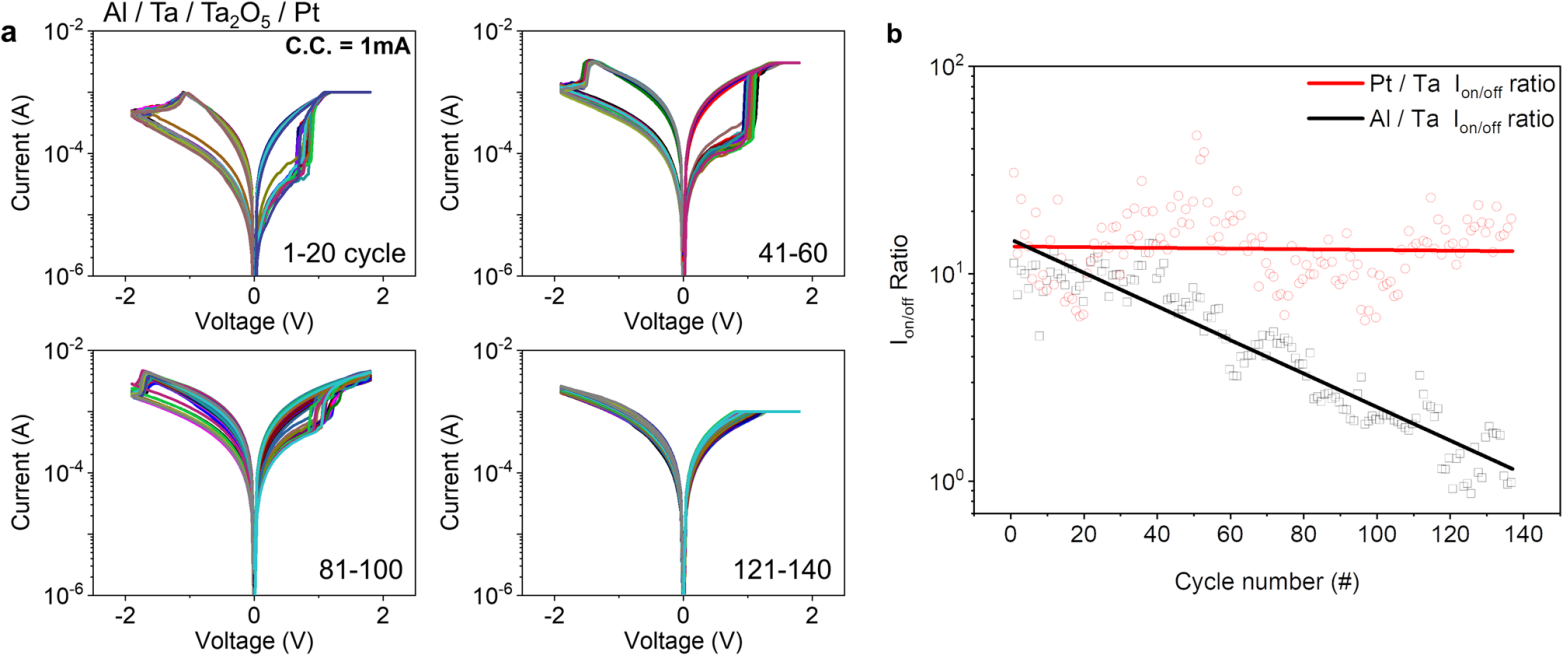
DC current–voltage characteristics and diagram of switching ratio change of Al and Pt Top electrode device (**a**) *I*–*V* characteristics of Al/Ta/Ta_2_O_5_/Pt device (**b**) On/off current ratio of each cycle measured at 0.3 V.

To support the CRS model based on modulation of Schottky barrier, band structure of Ta_2_O_5_-based ReRAM was decided by measuring transmittance spectra and ultraviolet photoelectron spectroscopy (UPS), which can provide optical bandgap and Valence Band Maximum of oxide films. Three tantalum oxide samples having different oxygen concentration were prepared using RF sputtering: Ta_2_O_5_ film (100 nm) from a Ta_2_O_5_ ceramic target and two oxygen deficient films (50 nm) by using reactive sputtering of Ta metal target with different oxygen pressure. Details can be seen in Table 1. The optical bandgap, ${E}_{g}^{opt}$, can be extracted from the transmittance spectra result in Fig. 4a, b using equations as follows:

1 $$ a = \frac{1}{d}{\ln}\left( \frac{1}{T} \right), $$

2 $$ \left( {ahv} \right)^{m} = A\left( {hv - E_{g} } \right), $$

where $a$ is an absorption coefficient calculated by film thickness $d$ and measured transmittance $T$. And in Eq. (2), $h$ and $v$ are Planck’s constant and photon frequency in Tuac relation^24^. In the fitting, we assumed that Ta_2_O_5_ and sub-oxide TaO_x_ are a direct bandgap material ($m$=2)^25^. In addition, to complete the band structure, Valence Band Maximum (VBM) was also measured using UPS results in Fig. 4c and the electron affinity $\chi$ is calculated from the bandgap and VBM. We used reported work function values for metals, Pt (5.65 eV), Ta (4.25 eV), and Al (4.28 eV)^26^. The results are summarized. From the measured band structure date, the fabricated device is expected to have a Schottky barrier at the Ta_2_O_5_/Ta (TE side) and Pt/Ta_2_O_5_ (BE side) interface as shown in Fig. 4d, which decides the HRS current level without conductive metallic filament. However, when changing the Ta film to tantalum sub-oxide by reactive sputtering, the barrier between TaO_x_ and Pt is believed to be manifest at the TE side interface and the barrier height would increase as oxygen concentration grows as described in Fig. 4e, f; 2.65 eV and 2.99 eV of energy height for low and high O_2_ concentration case respectively. This result well-explains changing of Schottky barrier by different oxygen concentration, but insufficient to prove the model directly. To further support the proposed model with more convincing way, various conduction mechanisms were tested to fit the BRS and CRS *I*–*V* curves as shown in Fig. 5. As expected, the linear *I*–*V* curve was found in double-logarithmic plot in the LRS at the BRS mode (1 mA C.C.) (Fig. 5b), which means metallic conduction. In contrast, at the CRS case (3 mA C.C.), we confirmed non-linear curve (Fig. 5d) and two main conduction mechanisms, tunneling and Schottky emission, were revealed from the *I*–*V* curve^7,27^. Especially in the high electric field, Schottky emission was the main electron conduction mechanism indicating presence of the Schottky barrier. In addition, from a temperature measurement, it was observed that the conduction mechanism changes from metallic conduction to Schottky emission with increased programing voltage, indicating switching mode change from BRS to CRS due to Schottky barrier, and the thermal activation energy of Schottky emission was 0.17 eV (Supplementary Fig. S2). From the above results, when positive bias is applied to the device, oxygen vacancies migrates toward BE along electric field, while oxygen ion movement takes opposite direction to Ta layer. And the oxygen ions may react with Ta metal at the top electrode and ends up forming a tantalum sub-oxide. This process is expected to be more obvious at higher voltage and current due to larger ion migration. Thus, with higher C.C. level, TaO_x_ with higher oxygen concentration will be formed under positive bias and this leads to elevation of Schottky barrier height at the TE interface as shown in Fig. 4f, finally causing turning-off of the device despite of the complete conductive filament in Ta_2_O_5_ layer in the set operation. In addition, the process depends on Ta thickness as well. Because the oxidation may not be a local process only at the interface of Ta_2_O_5_ and Ta, but it is rather believed to occur in a wide range of Ta layer even at the Ta and Pt interface^23^. Hence, a thicker Ta film may work to reduce the effective oxygen concentration of formed TaO_x_. As a result, the CRS behavior becomes less dominant with thicker Ta. The claim of wide-ranging oxidation may be partially supported by measured switching behavior of Al electrode device in Fig. 6a. Here, we only changed Pt to Al in TE, maintaining other layers from Fig. 1a. In the cycling test, Al TE device shows stable switching without CRS due to low 1 mA C.C. in the early stage. However, it rapidly degrades resistive switching properties and becomes not switching after around 120 cycles. On/Off ratio of Al TE device shows the degradation, which is not observed in Pt device (Fig. 6b). This may attribute to the reaction of Al electrode with oxygen ions arrived across the Ta layer and the resulting AlO_x_ may cause difficulty in resistive switching due to higher binding energy^28^. Interestingly, the degradation of the Al device still happens in thicker Ta (50 nm) case. Thus, it is claimed that the oxygen ion can spread out wide range of Ta layer during set operation and can form tantalum sub-oxide even at the interface with inert Pt electrode. This in turn leads to modulation of Schottky barrier at TE interface and results in CRS behavior at higher C.C. in thin Ta devices.

**Table 1:** Sample condition and measured values of optical band gap, VBM, and work function.

Oxide	Sputtering process		Optical band gap (eV)	VBM (eV)	Work function (eV)	Schottky barrier height with TE (eV)
	Target	Ar/O (sccm)				
Ta_2_O_5_	Ceramic	20/0	3.95	1.78	3.55	2.88 (Ta)
TaO_x_ (low)	Metal	20/3	2.75	1.42	4.32	2.65 (Pt)
TaO_x_ (high)	Metal	20/6	2.75	1.12	4.30	2.99 (Pt)

Schottky barrier height with Top Electrode is calculated.

## Conclusions

In summary, we experimentally showed reversible change of switching mode between BRS and CRS in Ta_2_O_5_ ReRAM device by controlling C.C. level and thickness of reactive metal electrode. The first time a thick electrode was used, showing BRS behavior regardless of the C.C. change. Second, in the same structure but with thinner Ta film, BRS was maintained at low C.C., while CRS appears at high C.C. The switching mode change was reversible according to C.C. level. The mechanism of CRS was studied using optical and electrical measurements and it is believed that Schottky barrier height at the TE interface is modulated depending on the oxygen amounts: the larger oxygen concentration, the higher barrier. The elevated barrier turns off ReRAM despite of existing conductive filament, and causes CRS type especially in high C.C. and thin Ta device. The result is useful for designing stable BRS or CRS device.

## Methods

### Device fabrication

The bottom electrode was formed by photo-lithography and lift-off process combining with radio-frequency (RF) sputtering of Ti and Pt on commercial SiO_2_/Si. Following Ta_2_O_5_ oxide, resistive switching layer, was deposited by RF sputtering with a Ta_2_O_5_ ceramic target. After that, Ta or Al and Pt electrodes were also formed by RF magnetron sputtering and photo-lithography process.

### Characterizations

DC electrical measurements were performed using a Keithley 4200 semiconductor parameter analyzer (Keithley 4200 SPA, Keithley Instruments, Inc.). Measurements were performed in air. The optical band gap was measured using Ultraviolet–visible (UV–Vis) transmittance was characterized with PerkinElmer Lambda 35 Ultraviolet–visible spectrophotometer from 200 to 800 nm at the interval of 1 nm. The work function of each electrode and electrode-pentacene interface was measured using ultraviolet photoelectron spectroscopy (UPS) (PHI 5000 VersaProbe(Ulvac-PHI, Japan)).

## Supplementary information

Supplementary information. (DOCX 193 kb)
