# Supplementary material for: Reversible switching mode change in Ta2O5-based resistive switching memory (ReRAM)
Source: Sci Rep. 2020 Jul 9;10:11247. doi: 10.1038/s41598-020-68211-y (PMC7347604; doi:10.1038/s41598-020-68211-y)
Supplement: Supplementary file 1 — Supplementary information. (DOCX 193 kb) [file 41598_2020_68211_MOESM1_ESM.docx]

SUPPLEMENTARY INFORMATION

Correspondence and requests for materials should be addressed to J.Y ([jeongyeonjoo@kist.re.kr](mailto:jeongyeonjoo@kist.re.kr))

**Reversible switching mode change in Ta_2_O_5_-based resistive switching memory (ReRAM)**

Taeyoon Kim^1^, Heerak Son^1^, Inho Kim^1^, Jaewook Kim^1^, Suyoun Lee^1^, Jong Keuk Park^1^, Joon Young Kwak^1^, Jongkil Park^1^ and YeonJoo Jeong^1,*^

**1. Schematic illustration of Complementary Resistive Switch (CRS) process**


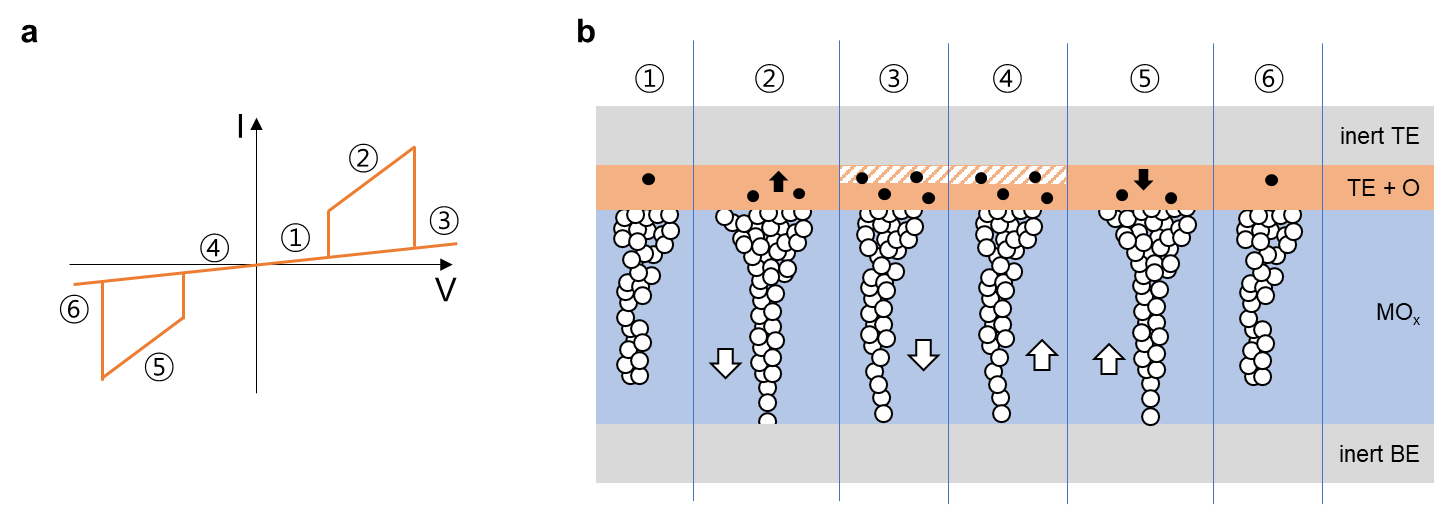


**Figure S1** (a) I-V curve of a common CRS device. (b) Schematic illustration of complementary resistive switching processes in a device having structure: inert top electrode (TE) / active top electrode / metal oxide / inert bottom electrode (BE). The directions of ion migration, including oxygen vacancies (white circle) and oxygen ions (black circle), are represented by the arrows. Six states in (b) correspond to the states in (a)

When positive bias is applied to the top electrode (TE) of the device, oxygen vacancies migrates toward bottom electrode (BE) along electric field, while oxygen ion movement takes opposite direction to Ta layer. And the oxygen ions may react with Ta metal at the TE and ends up forming a tantalum sub-oxide. This process is expected to be more obvious at higher voltage and current level due to the migration of more ions. Thus, with higher C.C. level, TaO_x_ with higher oxygen concentration will be formed under positive bias and this leads to elevation of Schottky barrier height at the TE interface, finally causing turning-off of the device (region 3 in Fig. S1) despite of the complete conductive filament in Ta_2_O_5_ layer in the Set operation. On the other hand, when a negative bias is applied to the device, the oxygen vacancies and oxygen ions move along the electric field as opposed to the positive bias case. As a result, the oxygen concentration of the tantalum sub-oxide decreases and consequently the current increases with the lowered Schottky barrier height (region 5 in Fig. S1). After that, the device faces Reset by rupturing of the conductive filament (region 6 in Fig. S1).

**2. Temperature-dependent resistance at different resistance states of Sample A**

**Figure S2** (a) CRS *I-V* curves of Pt / Ta(20nm) / Ta_2_O_5_ / Pt device. (b) Temperature-dependency of resistance was measured right after device turn-on (red circle) and metallic behavior was observed. (c) The same temperature test was done after reset process of CRS behavior (green circle) and (d) Activation energy (0.17eV) was extracted from the data.
